# *Fusarium* Community Composition and Co-Occurrence with Other Fungi and Bacteria in Finnish Oat Crop

**DOI:** 10.1007/s00248-026-02848-6

**Published:** 2026-08-03

**Authors:** Elina Sohlberg, Arja Laitila, Sari Rämö, Tuija Sarlin

**Affiliations:** 1 Process microbiology and food safety, VTT Technical Research Centre of Finland Ltd, Espoo, 02130 Finland; 2 Natural Resources Institute Finland (Luke), Jokioinen, 31600 Finland

**Keywords:** *Fusarium*, Cereal Microbiome, Microbial Community Structure, Community Interactions, Mycotoxins

## Abstract

*Fusarium* species are major contributors to Fusarium head blight (FHB) and mycotoxin contamination in cereal production. To characterize microbial co-occurrence associated with toxin accumulation in oats, the *Fusarium*, fungal, and bacterial communities of 51 Finnish oat grain samples were analyzed using a multi-marker metabarcoding approach targeting *Fusarium* translation elongation factor 1‑alpha (EF1α), fungal ITS2, and bacterial 16S regions. Microbial profiles were compared between grains with high and low deoxynivalenol (DON) concentrations. High‑DON samples were dominated by *F. graminearum*, whereas *F. langsethiae* prevailed in low‑DON grains. Bacterial diversity was higher in high‑DON samples, while fungal diversity remained similar across groups. Several bacterial genera, including *Pseudomonas*, *Paenibacillus*, *Massilia*, and *Curtobacterium*, exhibited negative correlations with *Fusarium* species, indicating competition in the same niche, whereas *Duganella* and *Xylophilus* showed positive association with *Fusarium* abundance. *Microdochium* consistently co-occurred with *Fusarium*. Results show differences in microbial communities associated with mycotoxin levels and *Fusarium* community composition, identifying taxa that may influence FHB or indicate mycotoxin risk.

**Graphical Abstract:**

**Figure:**
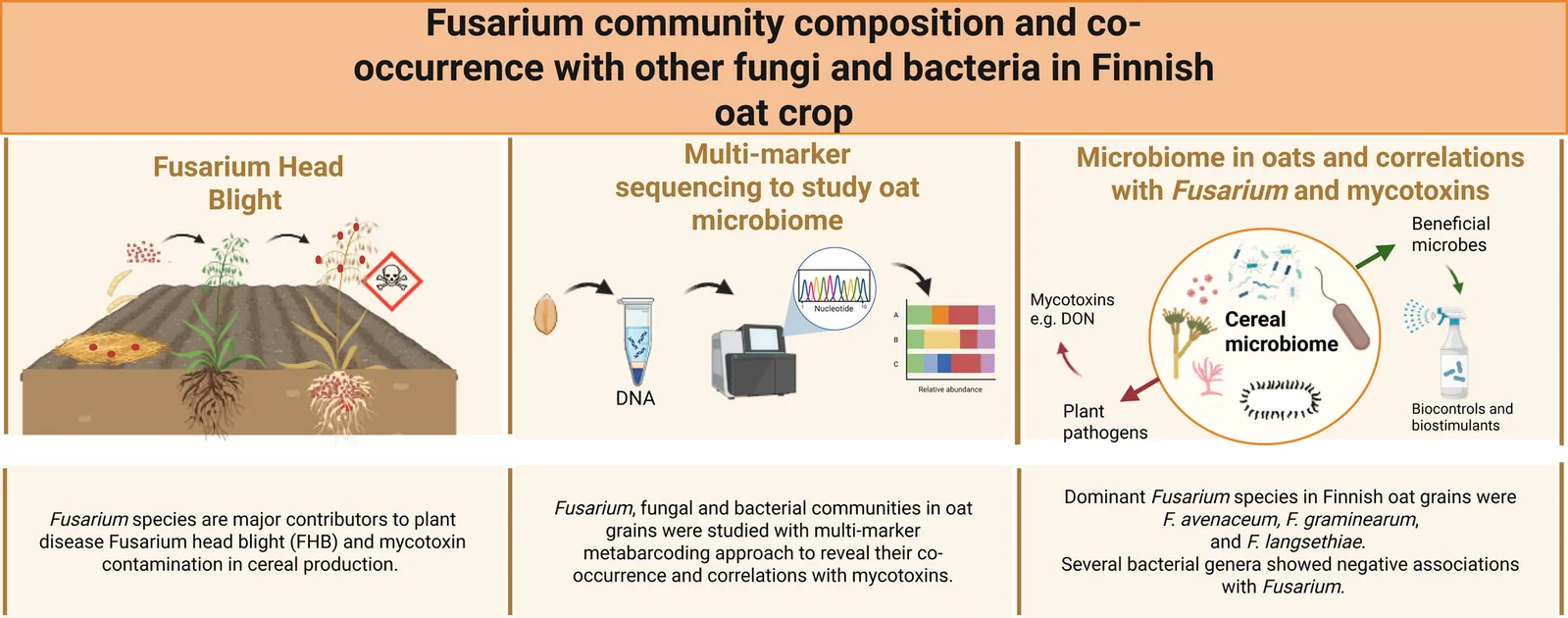

**Supplementary Information:**

The online version contains supplementary material available at https://doi.org/10.1007/s00248-026-02848-6.

## Introduction

Cereal crops, including oats, are globally crucial food and feed sources, yet they show high susceptibility to diseases associated with various plant pathogens [1]. Fusarium head blight (FHB), caused by species belonging to the genus *Fusarium*, represents one of the most destructive cereal diseases worldwide, reducing grain yield and quality, leading to contamination with harmful mycotoxins, and causing economic losses [2]. Current data show that up to 60–80% of crop samples contain detectable levels of mycotoxins, with 20–25% of global crops exceeding regulatory thresholds [3]. FHB poses a significant risk in northern agricultural regions, where cool and humid conditions favor infection and toxin production [4, 5].

FHB in oats is associated with several *Fusarium* species that differ in ecological niches and mycotoxin profiles, including *F. graminearum*,* F. avenaceum*, *F. langsethiae*, and *F. poae* in Northern Europe [6, 7]. In addition, the closely related plant pathogens *Microdochium nivale* and *M. majus* can cause FHB symptoms. Rather than a single species causing the disease, *Fusarium* species form a disease complex where different *Fusarium* species co-occur in the infected cereals [8]. The most significant mycotoxins produced by *Fusarium* plant pathogens include trichothecenes, such as deoxynivalenol (DON), nivalenol (NIV), T-2, and HT-2, and zearalenone (ZEN). Across Nordic countries, the dominant *Fusarium* species in oats vary by region but typically include *F. graminearum*, *F. avenaceum*, *F. langsethiae*, and *F. poae*. In Finnish oats, *F. graminearum* and *F. avenaceum* are most prevalent [9–12]. One of the most often detected mycotoxins in Finnish cereals is DON, produced primarily by *F. graminearum* [13]. DON is mainly produced by *F. graminearum* and by *F. culmorum*, both of which are major contributors to FHB-associated mycotoxin contamination in cereals.

Recent studies demonstrated changes in *Fusarium* populations in cereals, with previously rare species now emerging [14]. In the past two decades, a shift from *F. culmorum* to *F. graminearum* in FHB-infected cereals in Northern Europe has been documented [11, 15–17], and this trend is also evident in recent oat studies [9, 10, 12]. *Fusarium langsethiae*, the primary producer of T-2 and HT-2 toxins, dominates in colder climates in Northern Europe, and its ability to grow and produce mycotoxins at low temperatures has been demonstrated in in vitro studies [18]. In 2022, *F. langsethiae* was reported for the first time in oat grains in Spain [19] and previously in wheat in Italy [20], indicating its adaptation and movement toward areas with higher temperatures. Climate change and farming practices are cited as contributing factors to the emergence of new species [21]. These shifts highlight the need to characterize the entire *Fusarium* community in cereals to assess mycotoxin risk and target management strategies. Understanding shifts in *Fusarium* species is particularly important due to the differing mycotoxin profiles and associated risks among species. Because FHB involves a complex of interacting species rather than a single pathogen, characterizing the entire *Fusarium* community is essential for understanding disease dynamics and mycotoxin risk. Importantly, shifts in *Fusarium* species composition may also influence interactions within the surrounding microbial community, as different species vary in their ecological niches, colonization strategies, and mycotoxin profiles. These dynamics underline the importance of characterizing the broader microbial community associated with FHB to better understand factors influencing disease development and mycotoxin contamination.

Plant-associated microbiomes play a crucial role in plant health and in inhibiting pathogens. Cereal crops host diverse microbial communities, including bacteria, fungi, archaea, and viruses, which inhabit soil, plant surfaces, and internal tissues and can have beneficial, neutral, or detrimental effects on plant health [22]. Microbial communities vary across different parts of the plant, with the rhizosphere, i.e., soil directly surrounding plant roots, generally exhibiting greater microbial diversity compared to the internal tissues of plants (endosphere) or the above-ground surface area of plants (phyllosphere) [23]. Endophytic bacteria show specificity to certain plant genera, while epiphytic bacteria living on the surfaces of plants are common across many plant species [24]. Oat grains are commonly colonized by fungal genera such as *Fusarium*,* Cladosporium*, *Alternaria*, and *Microdochium*, as well as bacterial genera including *Pseudomonas*, *Pantoea*, *Massilia*, and *Sphingomonas* [22, 24]. Microbial taxa in grains originate from multiple sources, including the plant phyllosphere, the surrounding environment (e.g., air and soil), and earlier colonization stages during plant development [14, 25]. These microorganisms may colonize the grain before, during, or after plant pathogen infection, resulting in a complex microbial community within the infected tissue. Although the importance of the rhizosphere for plant health, fitness, resilience, and productivity has long been recognized, recent studies have also highlighted the role of the less abundant microorganisms colonizing aboveground plant tissues in host resilience and pathogen defense [14]. In the context of *Fusarium* head blight, interactions between *Fusarium* species and the surrounding microbial community may influence pathogen establishment, disease severity, and mycotoxin accumulation [14, 26].

*Fusarium* species interact with a diverse range of microbes throughout their life cycle in plant roots, stems, leaves, heads, or crop residues [26]. *Fusarium* infection typically occurs during flowering, when fungal spores infect cereal ears and colonize developing grains [5]. During this process, *Fusarium* species invade plant tissues, compete for nutrients, and produce mycotoxins, which can influence both plant physiology and the surrounding microbial community. Interactions between plant pathogens and associated microbiome are complex and affect both the spread of disease and the accumulation of mycotoxins in grain. For example, bacteria such as *Pseudomonas* and *Sphingomonas*, and certain fungi such as *Epicoccum* and *Sarocladium*, may inhibit *Fusarium* growth [27]. Furthermore, fungi like *Microdochium* commonly inhabit the same environment as *Fusarium* and are frequently observed together with FHB symptoms [11].

Fungicide performance against FHB and associated mycotoxin accumulation has been limited and variable [5]. Since the EU Farm to Fork Strategy aims to reduce the use and risk of chemical pesticides by 50% by 2030, new control methods are called for. Biocontrol products, especially those based on microbial consortia instead of individual species, offer a promising alternative strategy. Most existing cereal microbiome studies focus on the wheat microbiome, whereas the oat microbiome remains less studied [14]. Although some research on the microbiome composition of cereals has been conducted, interactions between species remain under-researched. More information is needed on the co-occurrence of *Fusarium* species with other microbes and the ecological mechanisms that control pathogen abundance and mycotoxin levels in grains.

This study characterizes the composition of *Fusarium*, bacterial, and fungal communities in Finnish oat grains using a multi-marker metabarcoding approach. In addition to characterization of the *Fusarium* complex in oats, the study aims to identify potential bacterial and fungal taxa that co-occur with FHB-associated *Fusarium* species. Furthermore, the correlation of DON toxin concentrations to microbial diversity and composition is examined. This work provides new insights into microbiome–pathogen co-occurrence in oats and helps lay the foundation for new strategies for disease monitoring and control.

## Materials and Methods

### Field Samples

A subset of 51 oat cereal samples was selected among the yearly crop survey samples collected around Finland by the Finnish Cereal Committee from 2015 to 2017 [12]. Trichothecenes (DON, Acetyldeoxynivalenol (3-AcDON), H-2/T-2, and NIV) were analyzed as described by Hietaniemi et al. [28] and Sohlberg et al. [12]. DON content detected in the cereal samples ranged from < 25 to 26,000 µg/kg, with a mean concentration of 4966 µg/kg [12]. The concentrations of toxins H-2/T-2, NIV, and 3-AcDON are presented in Table S1. Both samples with high and low DON contamination were selected for this study. Samples were categorized as low vs. high DON using 1750 µg/kg in compliance with the amended Commission Regulation (EC) No 2023/915 threshold [29]. DON concentration varied between 0 and 1700 µg/kg (mean 854) in low DON samples (*n* = 16) and between 1800 and 26,000 (mean 6846) µg/kg in high DON samples (*n* = 35).

### DNA Isolation

DNA from ground cereal samples was extracted using the FastDNA^®^ SPIN Kit for Soil (MP Biomedicals, Ohio, USA). Cereal samples (10–20 g) were ground into flour using an IKA A11 basic analytical mill (IKA-Werke GmbH & Co., Baden-Württemberg, Germany). DNA extraction was performed on 100 mg of grain flour following the manufacturer’s instructions, with a modified lysis step: 2 × 1 min at 6.0 m s⁻¹ using the FastPrep^®^ Cell Disrupter and Lysing Matrix A tubes, with cooling on ice between runs. DNA was extracted in triplicate (technical replicates from the same homogenized grain sample) and stored at − 20 °C until analysis. The quality and quantity of extracted DNA were assessed using a NanoDrop 2000 spectrophotometer (Thermo Fisher Scientific, USA).

### Fungal, Bacterial and *Fusarium* Community Analysis with NGS

#### Sequencing and Library Preparation

DNA elutions extracted from 51 oat samples were sent to Illumina Miseq sequencing of the *Fusarium* translation elongation factor 1‑alpha (EF1α) region and the fungal ITS2 region to Microsynth AG, Switzerland. First, *Fusarium* EF1α libraries were sequenced with Nextera (Illumina, Inc., San Diego, CA, USA) two-step PCR with primers Fa-150 5′ CCGGTCACTTGATCTACCAG-3′ and Ra-2 5′ATGACGGTGACATAGTAGCG-3′ [30] and fungal ITS2 region (300–400 bp) with primers ITS3/ITS4 (ITS3: GCATCGATGAAGAACGCAGC, ITS4: TCCTCCGCTTATTGATATGC) [31]. Due to the phylogenetic proximity of *Fusarium* and *Microdochium*, EF1α primers are known to amplify both genera [12]. The libraries were sequenced at Microsynth AG using 2 × 250 bp paired-end sequencing on the MiSeq platform with the MiSeq v3 Reagent Kit. Negative controls for amplicon barcoding PCR and positive mock control, including the known amount of DNA from species belonging to the genus *Fusarium* (*F. culmorum* (D-80148), *F. graminearum (*D-95470)), and a few other fungal species isolated from the cereal matrix (*Cochliobolus sativus* (D-76039), *Trichoderma atroviride* (D-091338), *Penicillium verrucosum* (D-99750), *Aspergillus ochraceus* (D-00808)) were included in the mock control set. The bacterial 16S V3-V4 region from the same oat samples was sequenced at Eurofins (Eurofins Genomics, Konstanz, Germany) using primers 357 F (TACGGGAGGCAGCAG) [32] and 800R (CCAGGGTATCTAATCC) [33]. The bacterial libraries were sequenced with 2 × 300 bp paired-end sequencing on the Illumina MiSeq platform.

#### Bioinformatics Workflow

The sequence reads obtained from Illumina Miseq sequencing were analyzed using the DADA2 software package version 1.38.0 [34] and the DADA2 Pipeline Tutorial 1.16, with some modifications. DADA2 pipeline was run in RStudio (RStudio, Boston, MA, USA, version 2024.12.0 Build 467) with R version 4.4.2. First, the sequences were pre-filtered to remove ambiguous bases (Ns) that could affect accurate mapping. Then, primers were identified from the sequences and removed using the cutadapt tool [35]. The quality of the sequence reads was checked according to the DADA2 workflow. Next sequences were filtered and trimmed using the DADA2 filterAndTrim function. Filtering parameters maxN = 0, maxEE = c(2, 2), truncQ = 2 were used. A minimum length of 50 bp was used for fungal ITS data to remove spurious, very low-length sequences, which follows the standard DADA2 ITS workflow, which accounts for the high variability in sequence length of ITS regions. For *Fusarium* EF1α sequences, truncLen (225,215) and for bacterial 16S sequences truncLen (260,200) was used. The maximum possible error rates were calculated using the learnErrors command. Identical reads were de-replicated (unique sequences). Amplicon sequence variants (ASVs) were identified from the sequence data using the DADA2 pipeline’s core sample inference algorithm. Denoised paired reads were merged according to the DADA2 pipeline, and an ASV table was constructed. Subsequently, chimeric sequence reads were removed from the dataset using the removeBimeraDeNovo function with the consensus option. Finally, taxonomy from the domain to the genus level was assigned to ASVs using DADA2’s native implementation of the naive Bayesian classifier.

Taxonomy to genus level for fungi was assigned against the UNITE ITS reference taxonomy database version sh_general_dynamic_18.07.2024 [36] and for bacteria against the 16S Silva reference taxonomy version 138 [37]. Taxonomy to species level for *Fusarium* was assigned using an updated in-house-generated *Fusarium* EF1α reference database (version 18.10.2023) [38]. *Fusarium* EF1α metabarcoding data were derived from the same sequence dataset as in a previous study [11] but were reprocessed and reanalyzed in this study using an updated bioinformatics pipeline and the updated EF1α reference database. The ASV count tables and taxonomy tables for bacterial (16S), fungal (ITS), and *Fusarium* EF1α datasets are provided as supplementary material (Table S2).

All figures generated from the sequencing data were constructed with R using the phyloseq package [39] and ggplot2 [40]. Raw sequencing reads have been deposited in the European Nucleotide Archive (ENA) under project accession PRJEB47809. Individual 16S sequences are available under accessions ERR16749566–ERR16749616, ITS sequences under accessions ERR16749703–ERR16749753, and the *Fusarium* EF1α gene region sequences under accession numbers ERS7652901-ERS7652951.

#### Statistical Analysis

To investigate differences in microbiota composition between high- and low-DON groups, the phyloseq R package was used to calculate ASV Richness (Observed), estimated richness (Chao1), and diversity (Shannon and Simpson) within samples. Alpha-diversity measures were calculated from raw read counts with the estimate_richness function in phyloseq. Alpha diversity analyses of fungal communities were performed using the ITS dataset. Pairwise Wilcoxon rank-sum tests were performed to assess significant differences in alpha diversity between oat samples with high and low DON contamination.

Differences in community composition (β-diversity) among samples from high and low DON groups were evaluated using non-metric multidimensional scaling (NMDS) based on Bray–Curtis dissimilarity. Bray–Curtis dissimilarities were calculated from ASV-level count tables after subsampling to a common sequencing depth of 37,013 reads for the ITS dataset, and 13,425 reads for the 16S dataset using the *avgdist* function from the vegan R package. The significance of differences in community composition was tested using permutational multivariate analysis of variance (PERMANOVA) implemented via the *adonis2* function in the vegan package, with 999 permutations. To verify the assumptions of PERMANOVA, homogeneity of multivariate dispersions was assessed using Levene’s test. To identify taxa contributing most strongly to differences between high- and low-DON samples, a distance-based redundancy analysis (dbRDA) was performed at the ASV level. Model coefficients (loadings) were subsequently visualized at the genus level to improve interpretability using the miaViz package [41].

The qPCR-based quantification data of *Fusarium* spp., *F. graminearum*, and *F. culmorum* used in the correlation analyses was obtained from a previous study by Sohlberg et al. [12], based on the same oat grain samples. This quantitative data was used in the present study as part of the correlation analyses (Fig. 6) and is included in Table S1. Spearman’s correlations were performed to determine significant pairwise associations between FHB‑related parameters (mycotoxin concentrations, quantity of *Fusarium* sp. DNA (FusE), *F. graminearum* DNA, and *F.culmorum* DNA), and the relative abundance of the 30 most dominant bacterial and fungal genera observed in oats using the metabarcoding method. Cross-kingdom ASV correlations between high- and low-DON oat samples were calculated using Spearman’s correlation with the *cor.test* function in the psych R package. To further compare T‑2/HT‑2 toxin concentrations between samples with high and low DON contamination, differences between the two groups were assessed using a Wilcoxon rank‑sum test. All visualizations were conducted with the ggplot2 package, except cross-kingdom correlations were visualized with the *cor_plot* function in the psych package.

## Results

### Microbial Community Diversity

The microbial community analysis of the oat crop was conducted using metabarcoding. From bacterial metabarcoding, a total of 2,746,382 sequences, grouped into 1172 ASVs, were detected. The number of bacterial 16S sequences ranged from 13,415 to 122,964 (median 52,053). From the fungal analysis, a total of 4,254,497 sequences, grouped into 1105 ASVs, were detected. The number of fungal ITS sequences ranged from 37,013 to 152,953 (median 75,068). From *Fusarium* EF1α region sequencing, a total of 2,559,461 sequences were grouped into 723 ASVs, of which 496 ASVs belonged to the *Fusarium* genus. The sequence numbers from *Fusarium* EF1α sequencing ranged from 6005 to 132,904, with a median of 43,656. The most abundant ASV from EF1α data belonged to the genus *Microdochium*, which is a close relative of the *Fusarium* genus [42]. In addition to *Fusarium* and *Microdochium*, a smaller number of ASVs were assigned to other genera, such as *Trichoderma*. Species that did not belong to the *Fusarium* or *Microdochium* genera, which are associated with FHB, were extracted from the *Fusarium* EF1α data before studying the FHB community in more detail.

**Fig. 1:**
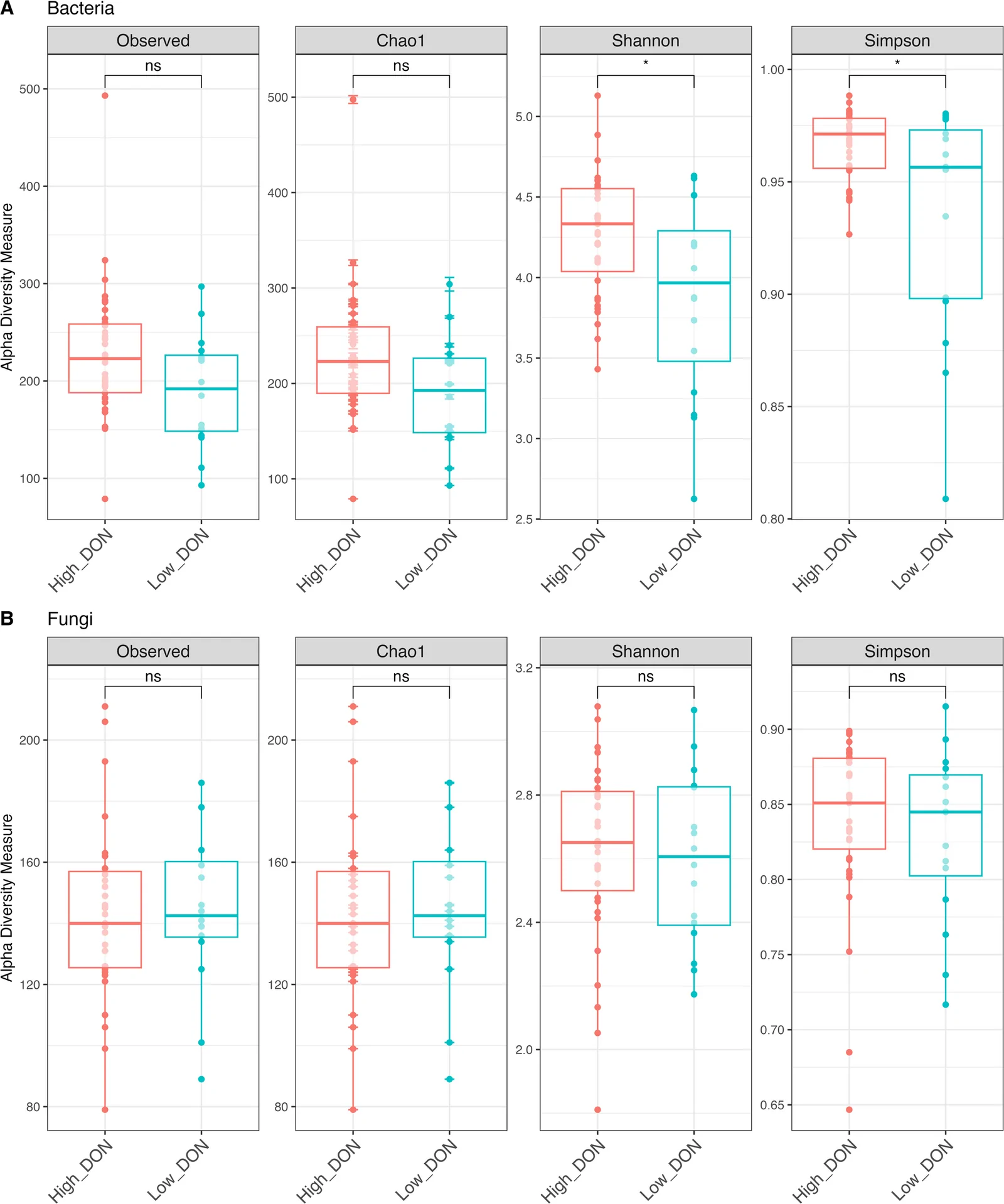
Alpha diversity of bacterial 16S (**A**) and fungal ITS (**B**) communities in oat grain samples with high DON and low DON concentrations. The Wilcoxon rank-sum test was used to compare the groups. ns = not significant, * *p*-value < 0.05

Alpha diversity analysis of the data revealed that the bacterial Shannon diversity and Simpson indices were higher (p-value < 0.05) in oat samples with higher DON levels (Fig. 1). The number of observed species and Chao1 were higher in high-DON samples, but the differences were not significant. The fungal alpha-diversity analysis showed that the number of observed species, Chao1, Shannon, and Simpson diversity indices were similar in both high- and low-DON samples.

### Microbial Community Composition

**Fig. 2:**
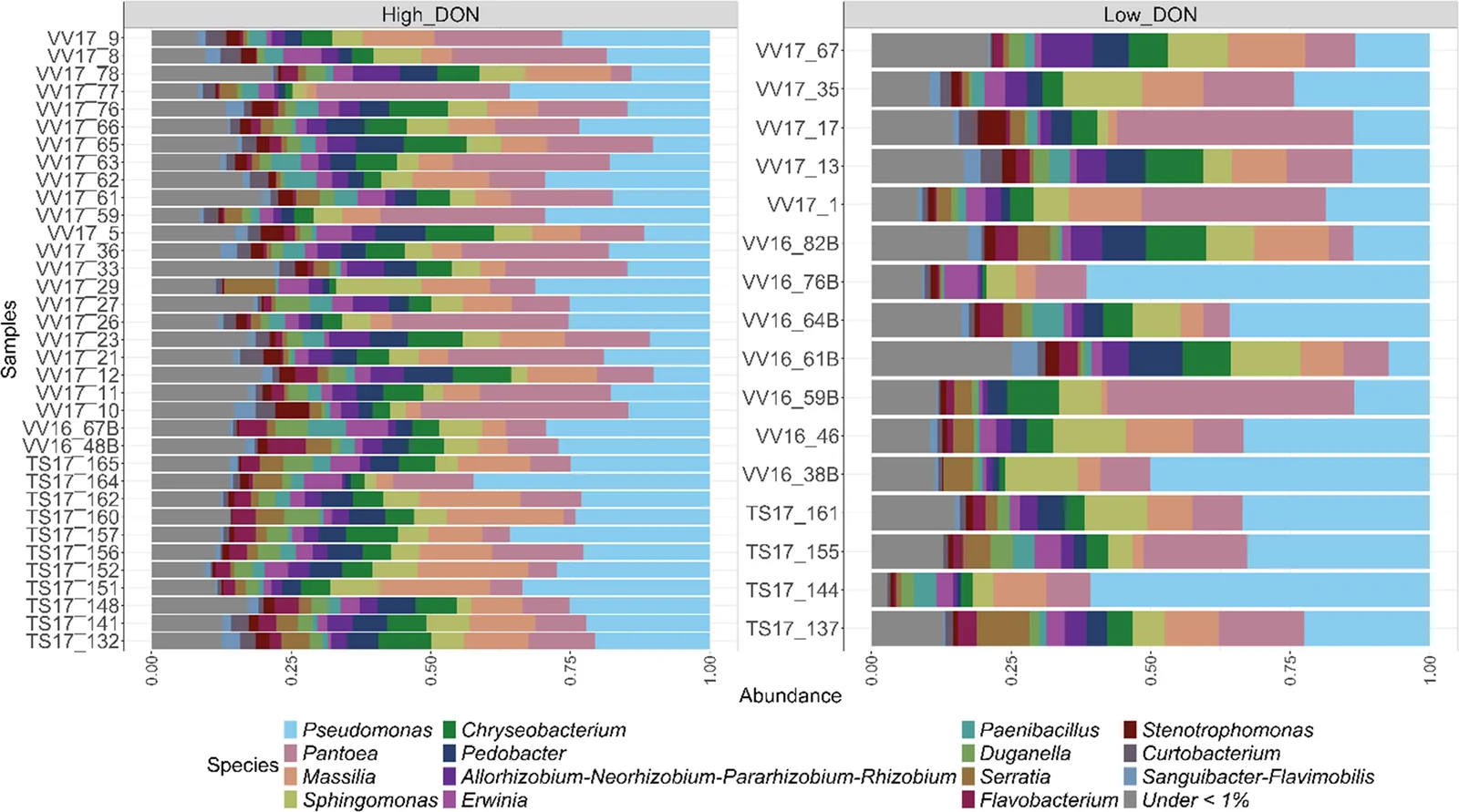
Relative abundance (%) of bacterial genera detected with the bacterial 16S metabarcoding method in the oat samples in high and low DON samples. Individual oat samples are shown and grouped according to DON level (high vs. low). Bacterial genera with relative abundance < 1% are grouped together as Under < 1%

The most abundant bacterial genera detected in Finnish oat crop were *Pseudomonas*, *Pantoea*, *Massilia*, and *Sphingomonas* (Fig. 2). The dominant bacterial genus, *Pseudomonas*, accounted for 23% (mean relative abundance) of the bacterial community in high-DON samples and 28% in low-DON samples. Another dominant bacterial genus, *Pantoea*, accounted for 16% of the bacterial community in both high- and low-DON samples. Genus *Massilia* and *Sphingomonas* accounted for 10% and 6% of the bacterial community in high-DON samples, and 8% in low-DON samples. Other minor bacterial genera (> 1%) detected in both sample types were *Chryseobacterium*,* Pedobacter*, *Allorhizobium*-*Neorhizobium*-*Pararhizobium*-*Rhizobium* group, *Erwinia*, *Paenibacillus*, *Duganella*, *Serratia*, *Flavobacterium*, *Stenotrophomonas*, *Curtobacterium*, and *Sanguibacter*-*Flavimobilis.*

**Fig. 3:**
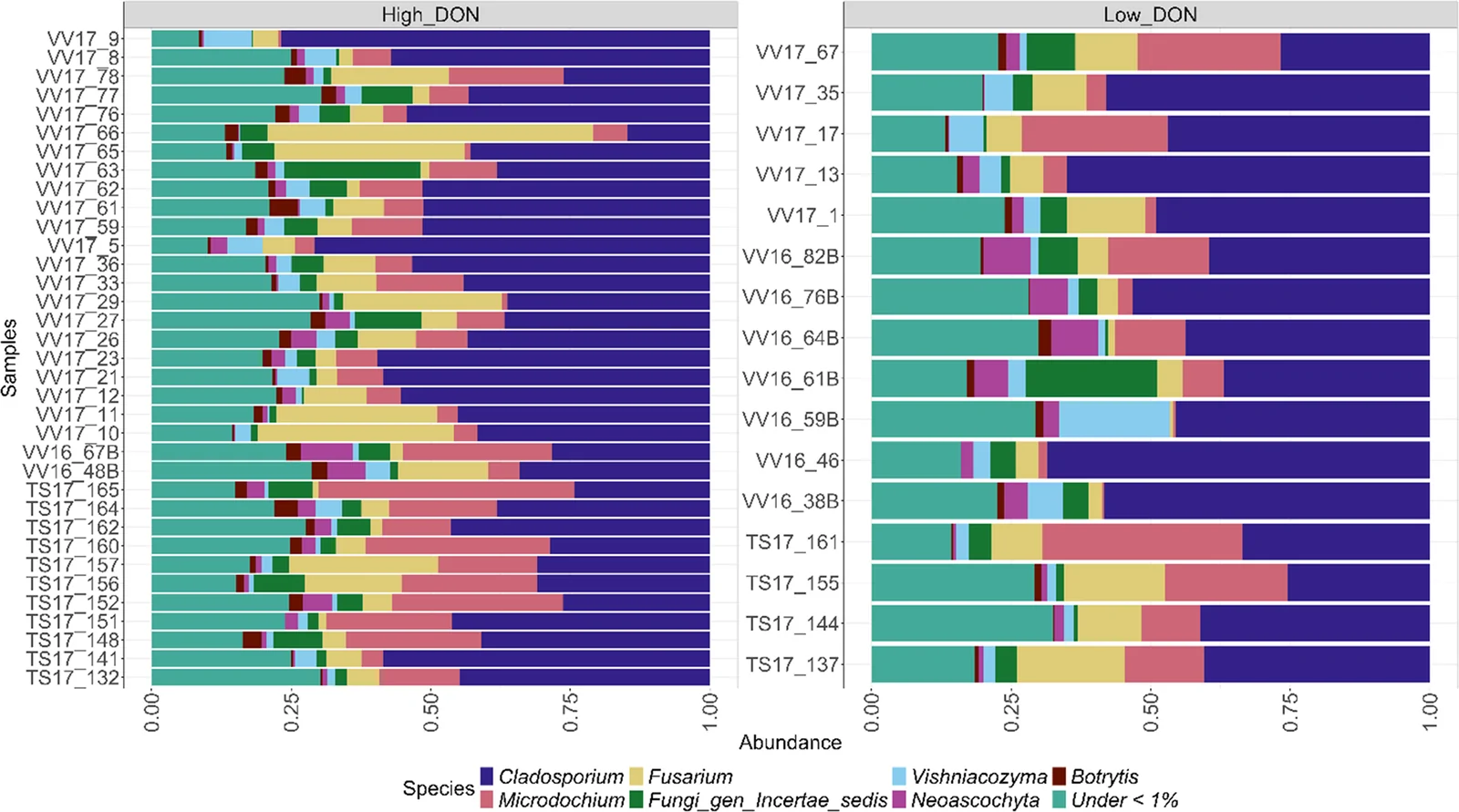
Relative abundance (%) of fungal genera detected with the fungal ITS metabarcoding method in the oat samples in high and low DON samples. Individual oat samples are shown and grouped according to DON level (high vs. low). Fungal genera under 1% relative abundance are grouped together as Under < 1%. Fungi_gen_Incertae_sedis represents fungal ASVs that could not be assigned to a known genus

The most abundant fungal genera in the Finnish oat crop were *Cladosporium*, *Microdochium*, and *Fusarium *(Fig. 3). The dominant fungal genus *Cladosporium* formed 44% (mean relative abundance) of the fungal community in high DON samples and 46% in low DON samples. Genus *Microdochium* comprised 13% of the fungal community in high DON samples and 12% in low DON samples. Genus *Fusarium* formed 11.5% of the fungal community in high DON samples and 8% in low DON samples. Other minor fungal (> 1%) genera detected in oat samples were *Vishiniacozyma*, *Neoascochyta*, and *Botrytis*.

**Fig. 4:**
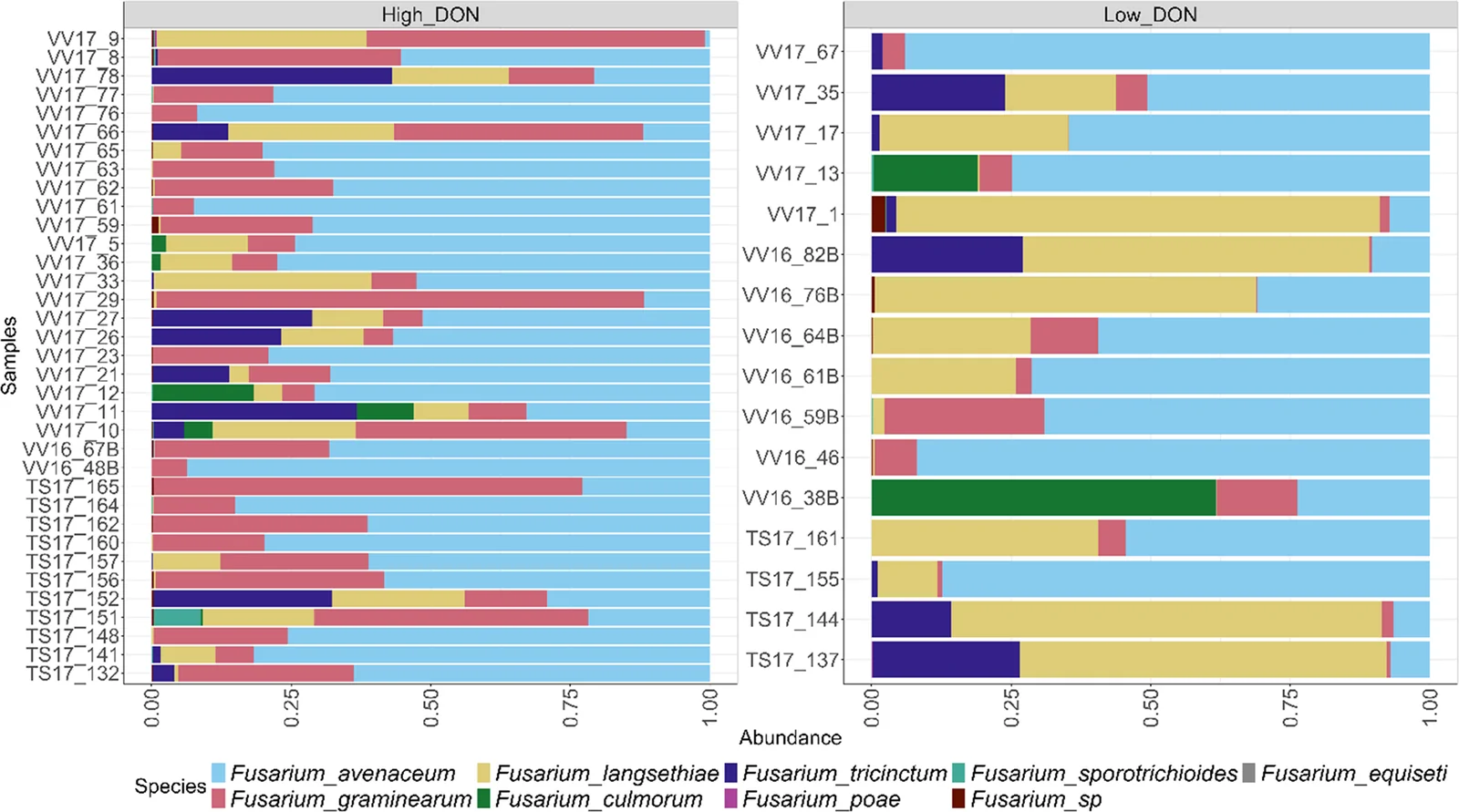
Relative abundance (%) of *Fusarium* species detected with the *Fusarium* EF1α metabarcoding method in high and low DON samples. Individual oat samples are shown and grouped according to DON level (high vs. low). The most abundant genus, *Microdochium*, was excluded from this figure to improve visualization of differences among *Fusarium* species

The most abundant *Fusarium* species detected in Finnish oat samples were *F. avenaceum*, *F. graminearum*, and *F. langsethiae* (Fig. 4). There were differences in *Fusarium* community structure between oat samples with high and low DON concentration. In both sample groups, the most common *Fusarium* species was *F. avenaceum*, with a mean relative abundance of 39% in high-DON samples and 35% in low-DON samples. *F. graminearum* was more abundant in high-DON samples (16%). In low-DON samples, *F. graminearum* was detected at a low level (4%). *F. langsethiae* was one of the dominant *Fusarium* species detected in low DON samples (24%), whereas in high DON samples its abundance was lower (6%). Other *Fusarium* species identified included *F. culmorum*, which comprised 4% of the *Fusarium* community in low-DON samples and 1% in high-DON samples. Similarly, *F. tricinctum* accounted for 5% of the community in low-DON samples and 4% in high-DON samples. Other minor *Fusarium* species (< 1%) detected were *F. sporotrichoides*, *F. poae*, *F. equiseti*, and *Fusarium* spp.

**Fig. 5:**
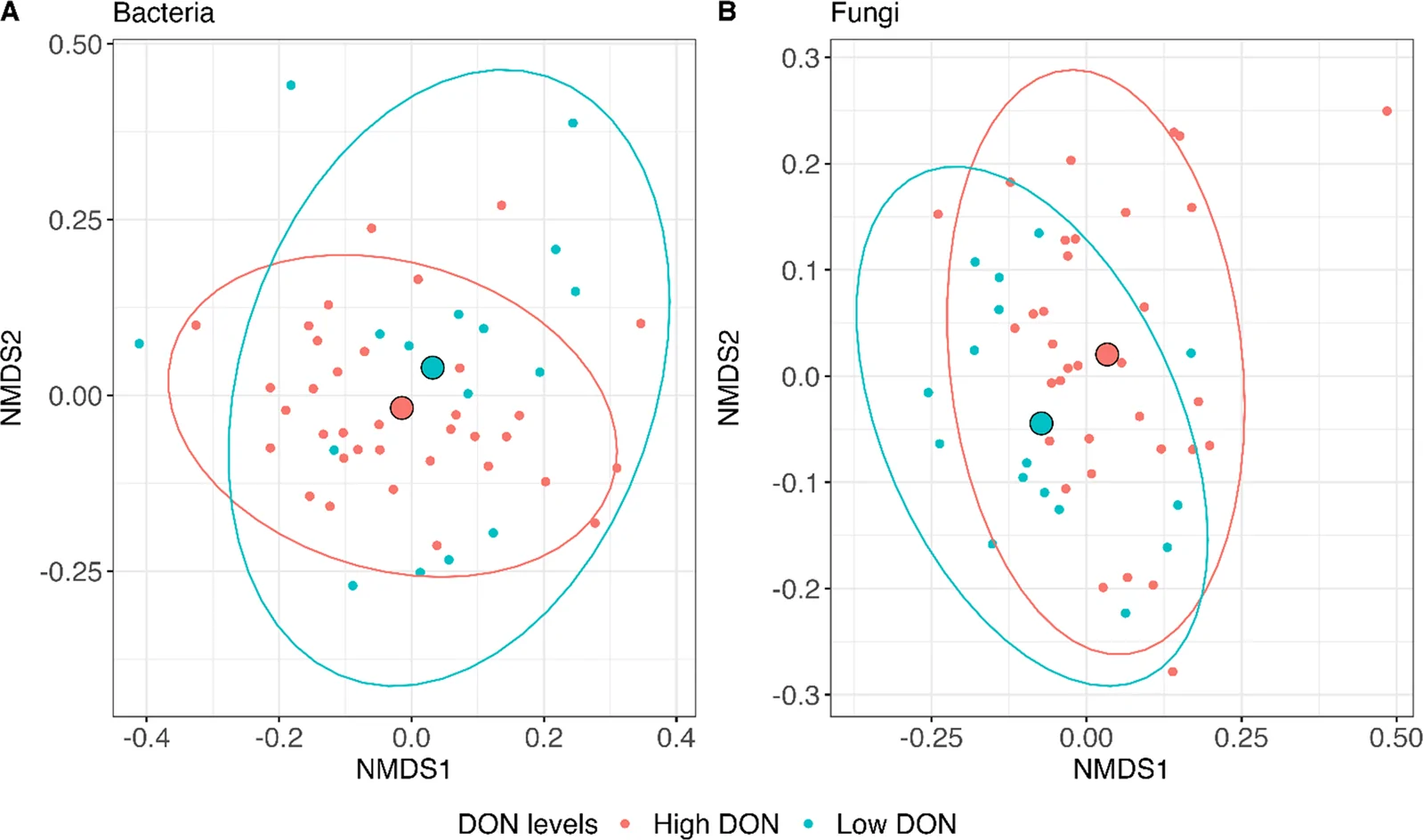
Fungal (**A**) and bacterial (**B**) community structure differences in high DON and low DON samples. A non-metric multidimensional scaling (NMDS) analysis of Bray–Curtis distances was performed to examine the relative abundances of fungal and bacterial ASVs. Prior to ordination, fungal (ITS) and bacterial (16S) datasets were subsampled to common sequencing depths of 37,013 and 13,425 reads per sample, respectively, using the avgdist function. Fungal communities differed significantly between DON groups (PERMANOVA: R² = 0.044, F = 2.28, *p* = 0.027; 999 permutations; stress = 0.198), whereas bacterial communities did not differ significantly (PERMANOVA: R² = 0.026, F = 1.33, *p* = 0.158; 999 permutations; stress = 0.203). The points represent individual oat grain samples, and ellipses indicate group dispersion. Larger points indicate group centroids (mean NMDS1 and NMDS2 coordinates)

Non-metric multidimensional scaling analysis of fungal and bacterial communities revealed differences between communities from oat samples with high and low DON concentrations (Fig. 5). High- and low-DON samples showed significant differences in fungal community structure (p-value = 0.027) in a PERMANOVA analysis. The betadisper result was not significant (Pr(> F) 0.9561), indicating that dispersion between groups is homogeneous and the PERMANOVA result is reliable. According to distance-based redundancy analysis (dbRDA), the fungal ASVs that exhibited the most significant differences between DON groups were *Fusarium* and *Cladosporium* sp. (Figure S1B).

High- and low-DON samples did not differ significantly in bacterial communities (p-value = 0.158 from adonis2). The betadisperser result was Pr(> F) 0.05632. The bacterial ASVs that differed mostly between DON groups were *Sphingomonas* spp. and *Pseudomonas* spp. according to the dbRDA analysis (Figure S1A).

### Correlation of Mycotoxins and *Fusarium* Abundance in the Oat Microbiome

**Fig. 6:**
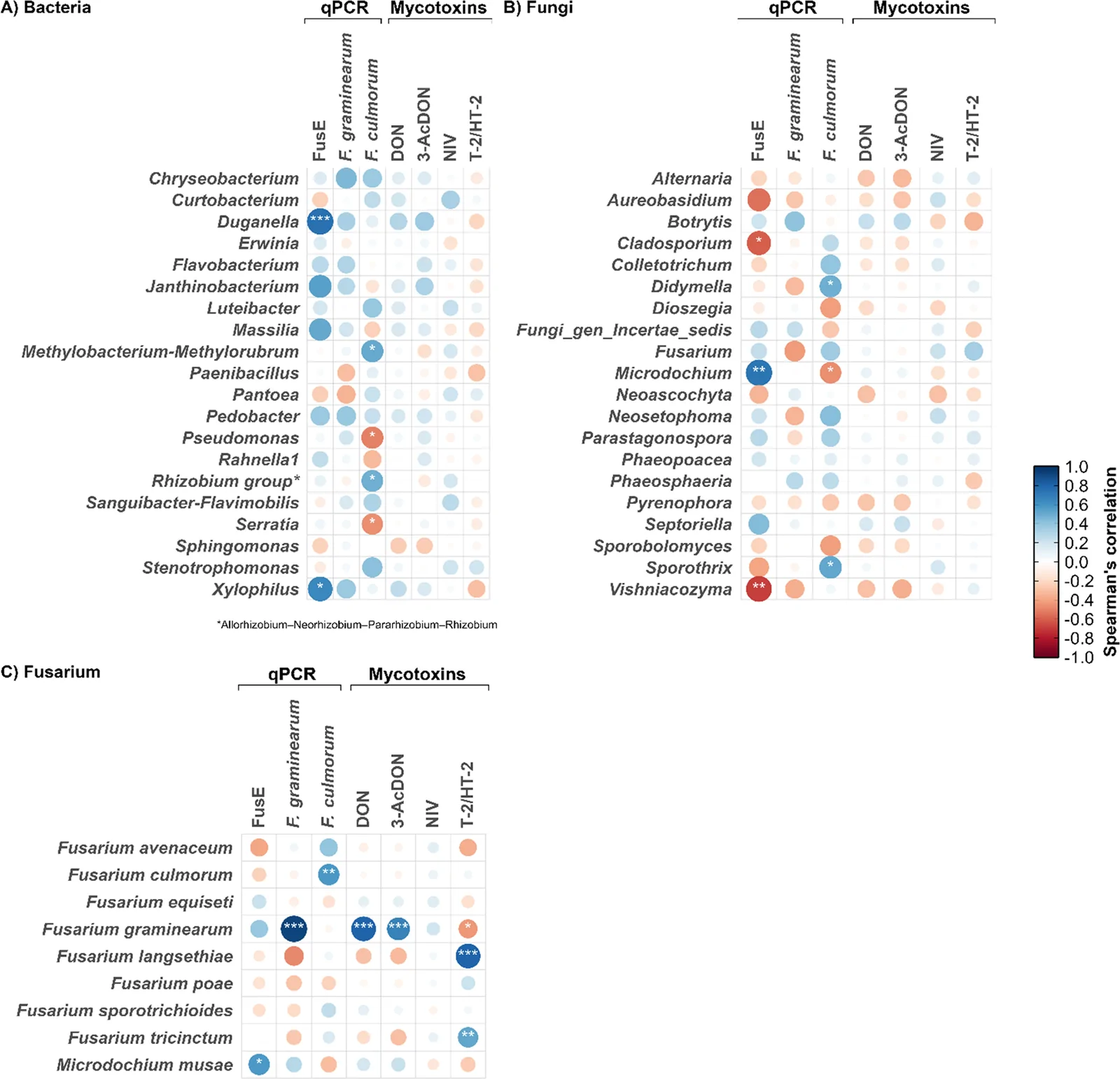
Significant pairwise associations among FHB-associated parameters: mycotoxin concentrations (DON, 3-AcDON, NIV, T-2/HT-2), quantities of Fusarium spp. DNA, F. graminearum DNA, and F. culmorum DNA determined by qPCR, and the relative abundances of the 20 most dominant bacterial (A) and fungal (B) genera, as well as nine detected Fusarium/Microdochium species (C) in oats, determined by metabarcoding. Associations were assessed using Spearman’s rank correlations. Red=negative, Blue=positive. FDR‑corrected p‑value thresholds are indicated as * p <0.05, ** p<0.01, *** p<0.001. Circle size indicates the strength of the correlation (absolute Spearman’s correlation coefficient)

A significant positive correlation between *Fusarium* sp. abundance (FusE qPCR) and the presence of bacterial genera *Duganella* (FDR < 0.001) and *Xylophilus* (FDR < 0.05) was detected (Fig. 6A). In addition, the *Rhizobium* group and *Methylobacterium*-*Methylorubrum* group had a significant (FDR < 0.05) positive correlation with *F. culmorum* abundance. *Pantoea*, *Sphingomonas*, and *Curtobacterium* negatively correlated with *Fusarium* abundance, whereas *Pseudomonas* and *Serratia* had a significant (FDR < 0.05) negative correlation with *F. culmorum* abundance.

*Cladosporium* and *Vishniacozyma* significantly (FDR < 0.01) negatively correlated with *Fusarium* sp. abundance measured with the FusE qPCR method (Fig. 6B). Other negatively correlated fungal species were *Aureobasidium*, *Neoascochyta*, and *Sporothrix*. *Microdochium* was significantly (FDR < 0.01) positively correlated with *Fusarium* sp. abundance but negatively correlated (FDR < 0.05) with *F. culmorum* abundance. *Didymella* and *Sporothrix* were positively correlated (FDR < 0.05) with *F. culmorum* abundance.

*F. graminearum* metabarcoding results significantly (FDR < 0.001) positively correlated with DON toxin, and *F. graminearum* abundance measured with qPCR (Fig. 6C), as expected. *F. graminearum* significantly (FDR < 0.05) negatively correlated with T-2/HT-2 toxin. *F. langsethiae* significantly (FDR < 0.001) positively correlated with T-2/HT-2 toxin and negatively correlated with *F. graminearum* abundance. Consistent with these correlations, T‑2/HT‑2 concentrations differed significantly between low‑ and high‑DON samples (Wilcoxon rank‑sum test, *p* < 0.001), with higher median levels in low‑DON samples. *F. tricinctum* significantly (FDR < 0.01) correlated with T-2/HT-2 toxin. Similarly, as in fungal data, *Microdochium* significantly (FDR < 0.05) positively correlated with *Fusarium* sp. abundance. 

### Co-Occurrence of Bacterial and Fungal Genera with *Fusarium* Species in Oat

Significant cross-kingdom correlations were observed between *Fusarium* species and associated bacterial and fungal taxa (Figure S2). A negative association was particularly evident between *Fusarium* (ASV19) and *Massilia*.

**Fig. 7:**
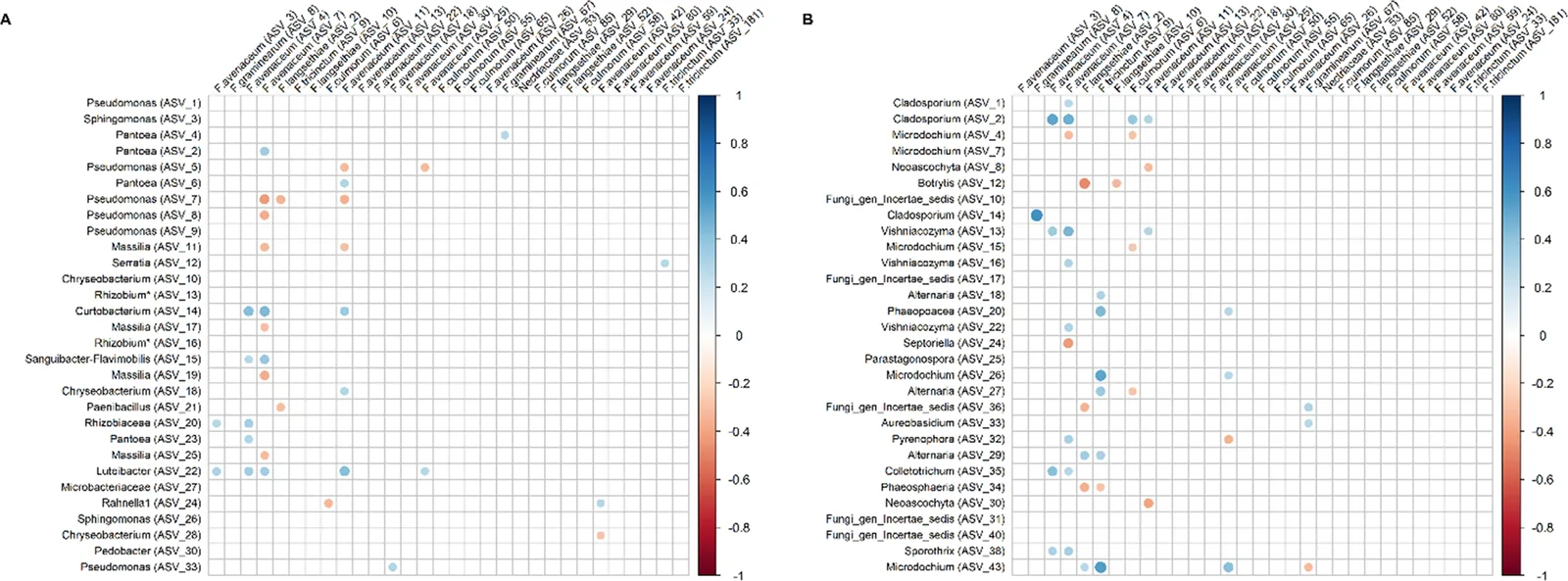
ASV‑level correlations between *Fusarium* and associated bacterial (**A**) and fungal (**B**) taxa in oat grains. Only the top 30 ASVs are shown, and only statistically significant correlations (Spearman’s rank correlation, *p* < 0.05) are displayed, with non‑significant correlations left blank. Panels A and B share the same color scale. Blue indicates positive correlations and red negative correlations; color intensity reflects the strength of the correlation coefficient. Rhizobium*= *Allorhizobium*-*Neorhizobium*-*Pararhizobium*-*Rhizobium*

Among bacterial taxa, several genera showed consistent negative correlations with *Fusarium* species. In particular, *Pseudomonas* ASVs (including ASV7) were negatively correlated with multiple *Fusarium* species, such as *F. avenaceum* (ρ = − 0.42, *p* = 0.002) and *F. langsethiae* (ρ = − 0.35, *p* = 0.01) (Fig. 7A). Similarly, *Paenibacillus* and *Rahnella* were negatively associated with *F. langsethiae* and *F. culmorum*, respectively. In contrast, *Rahnella* exhibited a positive correlation with *F. avenaceum* (ρ = 0.28, *p* = 0.04), indicating genus-specific interactions depending on the *Fusarium* species. Additional positive associations with *Fusarium* species were observed for *Luteibacter* (ρ = 0.42, *p* = 0.01) and *Curtobacterium* (ρ = 0.45, *p* = 0.001), while other positively associated genera included *Pantoea*, *Sanguibacter-Flavimobilis*, *Serratia*, and members of the *Rhizobiaceae*.

Among fungal taxa, several genera showed negative associations with *Fusarium* species. (Fig. 7B). For example, *Botrytis* was negatively correlated with *F. langsethiae* (ρ = − 0.48, *p* = 0.001), while *Phaeosphaeria* and *Neoascochyta* were also consistently negatively associated with *Fusarium* species. In contrast, the strongest positive associations were observed for *Cladosporium* and *Microdochium*. *Cladosporium* was positively correlated with *F. graminearum* (ρ = 0.61, *p* < 0.001), while *Microdochium* showed a strong positive correlation with *F. tricinctum* (ρ = 0.53, *p* < 0.001). Additional fungal genera positively associated with *Fusarium* species included *Vishniacozyma*, *Alternaria*, *Colletotrichum*, and *Sporothrix*.

## Discussion

This study characterized the *Fusarium*, bacterial, and fungal communities in Finnish oat grains using a multi-marker metabarcoding approach and assessed how these communities differ between grains with high and low levels of deoxynivalenol (DON). Microbial and *Fusarium* community composition and diversity patterns differed between samples with contrasting mycotoxin levels. Together, these findings improve understanding of the oat grain microbiome and highlight microbial taxa with potential roles in disease suppression, pathogen facilitation, or mycotoxin prediction.

A strong association between *F. graminearum* abundance detected by metabarcoding and elevated DON levels was observed, confirming its role as the dominant DON producer in Finnish cereals and supporting earlier findings by Parikka et al. [21] stating a shift from *F. culmorum* to *F. graminearum* in Finnish cereal crops. In contrast, *F. langsethiae* was more abundant in low‑DON samples and showed a strong positive correlation with T-2/HT-2 toxin levels, consistent with its known toxigenic profile in Nordic cereals [43]. This pattern was further supported by the significantly higher T‑2/HT‑2 concentrations observed in low‑DON samples compared with high‑DON samples, indicating contrasting mycotoxin profiles linked to differences in *Fusarium* species composition. The negative correlation between *F. langsethiae* and *F. graminearum* suggests competitive dynamics, with the more aggressive *F. graminearum* becoming dominant under conditions favorable to its growth and mycotoxin production. Previous in planta and in vitro studies with oat have shown that *F. langsethiae* is negatively affected when co-inoculated with *F. graminearum* and other *Fusarium* species [44]. In addition, *Fusarium* species differ in their environmental preferences, with *F. graminearum* primarily associated with humid conditions during anthesis, whereas species such as *F. langsethiae*, *F. sporotrichioides*, and *F. poae* are more strongly favored by warm conditions during the post-anthesis period [43].

The presence of *Microdochium* species, which consistently co‑occurred with *Fusarium* spp., offers insight into niche overlap among cereal pathogens. Their strong positive correlation with *Fusarium* abundance is consistent with earlier reports that *Microdochium* species are closely related to *Fusarium* and may co‑infect grain tissues or persist under similar environmental conditions [42, 45]. Both genera are also amplified with the *Fusarium*-specific EF1α primers, consistent with their close phylogenetic relationship [12]. However, earlier studies have also indicated negative correlations between *Fusarium* and *Microdochium* species [11, 46].

Research on oat microbiomes is still limited, even though oats are frequently contaminated by *Fusarium* mycotoxins and are associated with *Fusarium* species whose relative prevalence differs from that observed in wheat [47]. Previous studies report that the bacterial microbiome in oats consists of *Pseudomonas*,* Paenibacillus*, *Leuconostoc*, *Exiguobacterium*, *Rhizobium*, *Sphingomonas*, and *Stenotrophomonas* [48, 49]. The most abundant bacterial genera detected in the Finnish oat crop in this study were *Pseudomonas*,* Pantoea*, *Massilia*, and *Sphingomonas*. Several of the dominant bacterial genera identified in this study, including *Pseudomonas* and *Sphingomonas*, are commonly reported in cereal microbiomes and have been detected in wheat, barley, and oat across different geographic regions [30, 50]. These taxa are frequently associated with plant surfaces and grains and are known for their roles in plant–microbe interactions, including both beneficial and antagonistic effects on pathogens.

In this study, high DON levels were associated with increased bacterial α‑diversity without a corresponding shift in overall bacterial community structure. In contrast, fungal communities exhibited significant compositional differences between DON groups despite similar α‑diversity. Increased bacterial diversity in high-DON samples may reflect changes in the grain environment associated with *Fusarium* infection, such as tissue damage, altered nutrient availability, and the production of fungal metabolites, which can create additional ecological niches for microbial colonization [14, 26]. In addition, increased diversity may reflect the coexistence of opportunistic, biocontrol-associated, or stress-tolerant microorganisms that colonize grains under disease conditions. The bacterial community showed notable associations with *Fusarium* species and several genera, including *Pseudomonas*, *Sphingomonas*, *Paenibacillus*, and *Curtobacterium*, displayed negative correlations with *Fusarium* abundance. Many of these genera, especially *Pseudomonas*, are known producers of antimicrobial compounds or are capable of inducing plant defense responses [51, 52]. *Serratia* that showed a significant negative correlation with *F. culmorum* abundance in this study have been reported to suppress *Fusarium* species [53]. Similarly, negative correlations with *Paenibacillus* may relate to fusaricidin production, a known antifungal compound against *Fusarium* produced by the genus [54]. These associations suggest that the resident grain microbiota may contribute to limiting pathogen proliferation. However, such patterns may not necessarily reflect direct competitive interactions but could also arise from shared environmental conditions or host-associated factors. In contrast, the bacterial genera *Duganella* and *Xylophilus* co-occurred with *Fusarium* spp., suggesting either tolerance to the pathogens or the capacity to benefit from *Fusarium*‑altered substrates. These genera may serve as potential indicators of FHB presence or severity.

The major fungal genera in the Finnish oat crop in this study comprised *Cladosporium*,* Microdochium*, and *Fusarium*. Similar fungal genera have also been reported in oat tissues, which are commonly colonized by genera such as *Aspergillus*, *Alternaria*, *Cladosporium*, *Filobasidium*, *Fusarium*, *Mortierella*, and *Trichocladium* [18, 47]. Several ascomycete genera, including *Alternaria* and *Cladosporium*, are consistently reported as dominant members of cereal grain microbiomes across different studies and geographic regions [53, 54], reflecting their ability to colonize plant tissues under a range of environmental conditions. Their frequent detection suggests that they are core members of cereal grain microbiomes and may be involved in microbiome–pathogen associations.

Fungal genera detected from Finnish oat grains showed both negative and positive relationships with *Fusarium* species. Negative correlations with *Botrytis*, *Septoriella*, *Phaeosphaeria*, and *Neoascochyta* indicate possible competitive interactions within grains, given that these genera include plant pathogenic fungi capable of occupying similar ecological niches [55, 56]. The fungal genus that showed a significant positive correlation with *Fusarium* sp. was *Microdochium*, reflecting shared ecological niches or parallel colonization patterns. Conversely, *Cladosporium* showed mixed correlations across datasets, suggesting complex interactions that may vary with taxonomic resolution or environmental conditions. Similar patterns have recently been reported in wheat, where differences between high- and low-FHB conditions were associated with shifts in the mycobiome and with taxa potentially linked to pathogen suppression or facilitation, supporting the broader relevance of cereal microbiome–pathogen interactions across crop systems [57]. Negative correlations with *Fusarium* species may not solely reflect competitive interactions but can also be influenced by host-related or environmental factors, such as plant genotype or nutritional status [14, 26]. In addition, certain taxa may act as indicators of plant condition or infection status rather than directly interacting with *Fusarium* [27].

Overall, our findings illustrate that DON levels in oat grains are closely linked to changes in *Fusarium* community structure and to changes in cross‑kingdom associations within the grain microbiome. Taxa negatively associated with *Fusarium* may represent promising candidates for biocontrol, while positively associated taxa may serve as useful indicators of infection severity or toxin risk. These insights lay a foundation for targeted microbiome‑informed monitoring and management strategies for FHB in oats.

Although this study provides comprehensive insight into cross‑kingdom microbial interactions in Finnish oat grains, several limitations should be acknowledged. First, metabarcoding relies on DNA-based detection, which does not distinguish between active, dormant, or dead microbial cells and therefore may overestimate the ecological relevance of some taxa. Second, EF1α primers used for *Fusarium* metabarcoding also amplify *Microdochium*, complicating species-level resolution in these closely related genera despite subsequent filtering steps. In addition, the identification of the taxa is only as good as the reference database used. Third, metabarcoding data provide relative abundances rather than absolute quantities of organisms, which can limit direct comparisons with qPCR results. In addition, species-level identification is not always reliable when using short amplicons such as bacterial 16S and fungal ITS sequences amplified with universal primers. These methodological limitations may partly explain the lack of significant correlation between *Fusarium* ITS-based abundance estimates and qPCR results. Fourth, the study is based on field samples collected during three harvest years; although DON levels were clearly differentiated, environmental and agronomic factors could also have contributed to microbiome variation. Fifth, correlation-based analyses cannot demonstrate causality, and observed associations may partly reflect shared ecological niches rather than direct pathogen suppression or facilitation. Controlled experiments are needed to validate predicted microbial interactions and to assess the biocontrol potential of candidate taxa. Finally, although three genetic markers were used, shotgun metagenomics or metatranscriptomics could provide deeper functional insights into microbial interactions and toxin-related processes. In the future, longitudinal field studies and multi‑omics approaches would further clarify the mechanisms driving microbial interactions and improve the predictive value of microbiome‑based indicators. Such efforts will be essential for developing effective, sustainable strategies to reduce FHB pressure and mycotoxin contamination in cereal production systems. In addition to DON levels, factors such as oat variety and agronomic conditions may also influence microbiome composition and should be considered in future studies.

## Conclusions

This study demonstrates that deoxynivalenol (DON) contamination in Finnish oat grains is closely associated with differences in *Fusarium* species composition and with distinct cross‑kingdom microbial co‑occurrence patterns. High DON levels were associated with the dominance of *F. graminearum*. In contrast, low DON samples showed a higher relative abundance of *F. langsethiae*, reflecting differences in toxigenic potential and ecological niches. While fungal diversity remained relatively stable across oat samples with high or low DON contamination, bacterial diversity was higher in highly DON-contaminated samples, suggesting that mycotoxins and mycotoxin-producing fungi may influence bacterial diversity in oat grains. Several bacterial and fungal taxa showed consistent negative associations with *Fusarium*, suggesting potential competition within similar ecological niches, whereas positively associated taxa may serve as indicators of infection pressure or mycotoxin risk. These associations should be interpreted with caution, as they may reflect indirect effects such as host-related or environmental factors rather than direct microbial interactions. Together, these findings highlight the importance of considering whole‑microbiome interactions when assessing FHB and provide a basis for future microbiome‑informed monitoring and biocontrol strategies in oat production systems. Overall, our results suggest that *Fusarium* head blight in oats should be viewed as a complex system involving interactions between pathogen species, the associated microbiome, and environmental factors, rather than as a process driven by a single pathogen alone.

## Supplementary Information

Below is the link to the electronic supplementary material.

Supplementary Material 1

Supplementary Material 2

Supplementary Material 3

## Data Availability

Authors confirm that the data supporting the findings of this study are available within the article and its supplementary material. Sequencing raw data is available at the European Nucleotide Repository (ENA) at https://www.ebi.ac.uk/under project accession PRJEB47809 and the Fusarium EFα-sequence database in Mendelay Data (doi: 10.17632/wm8jr96vhh.1). Mycotoxin data and ASV and taxonomy tables are available as supplementary material.
