# Supplementary material for: Fusarium Community Composition and Co-Occurrence with Other Fungi and Bacteria in Finnish Oat Crop
Source: Microb Ecol. 2026 Aug 3;89:201. doi: 10.1007/s00248-026-02848-6 (PMC13737283; doi:10.1007/s00248-026-02848-6)
Supplement: Supplementary file 1 [file 248_2026_2848_MOESM1_ESM.docx]

**Figure S1**. dbRDA loadings showing bacterial 16S (A) and fungal ITS (B) ASVs with taxonomy on genus level that contributed most strongly to differences between the low‑ and high‑DON groups.

**Figure S2.** Cross-kingdom correlations between bacterial and fungal ASVs detected in oat grains. The top 30 ASVs from each dataset are shown, and only statistically significant correlations (Spearman’s rank correlation, p < 0.05) are displayed. ASVs are arranged according to genus-level classification while retaining ASV identifiers. Blue indicates positive correlations and red negative correlations; color intensity reflects the strength of the correlation coefficient. Non-significant correlations are left blank. ASVs classified as “Unclassified” on the genus level were excluded from the analysis.

**Table S1**. Mycotoxin (DON, 3-AcDON, NIV, T-2/HT-2) and qPCR (FusE, *F.graminearum*, *F.culmorum*) results used in correlation tests.

**Table S2.** ASV count tables and taxonomy tables for the 16S, ITS, and EF1α datasets. Each dataset is provided in a separate worksheet within the Excel file. Rows represent ASVs and columns represent samples.
